# Supernumerary nostril: a case report

**DOI:** 10.1186/s40902-016-0090-0

**Published:** 2016-11-05

**Authors:** Bo-Eun Choi, Seung-O Ko, Hyo-Keun Shin

**Affiliations:** Department of Oral and Maxillofacial Surgery, Chonbuk National University Hospital, 20, Geonji-ro, Deokjin-gu, Jeonju-si, Jeollabuk-do, 54907 Republic of Korea

**Keywords:** Supernumerary nostril, Accessory nostril, Congenital nasal deformities

## Abstract

**Background:**

Supernumerary nostril is a congenital anomaly that contains additional nostril with or without accessory cartilage. These rare congenital nasal deformities result from embryological defects. Since 1906, Lindsay (Trans Pathol Soc Lond. 57:329–330, 1906) has published the first research of bilateral supernumerary nostrils, and only 34 cases have been reported so far in the English literature.

**Case presentation:**

A 1-year-old female baby was brought to our department group for the treatment of an accessory opening above the left nostril which had been presented since her birth. Medical history was non-specific and her birth was normal. The size of a supernumerary nostril was about 0.2 cm diameter and connected to the left nostril. The right one was normal. Minimal procedure was operated for the anomaly. After 1 year, rhinoplasty was performed for the nostril asymmetry.

**Conclusions:**

At 1 year follow-up, the functional and cosmetic result was satisfactory. In this case, it is important that we have early preoperative diagnosis. Also, it is desirable that we should perform a corrective surgery as soon as possible for the patient’s psychosocial growth.

## Background

Supernumerary nostril is known as triple nostrils or accessory nostril, and also, it is a rare congenital anomaly. Only 34 reports of this literature were revealed since 1906 [1]. Supernumerary nostril was mostly unilateral and isolated. Also, it can be related with other congenital malformations such as a facial cleft. We report a case of female patient’s supernumerary nostril above the left nostril. As far as we know, this is the second report of accessory nostril in Korea.

## Case presentation

A 1-year-old female baby visited our department for the evaluation and treatment of an accessory opening above the left nostril which had been existed since her birth. The patient and her parents’ medical history was non-specific except that the patient had an accessory nostril. Also, the age of her mother was 32 years old and there was nothing wrong at parturition.

**Fig. 1:**
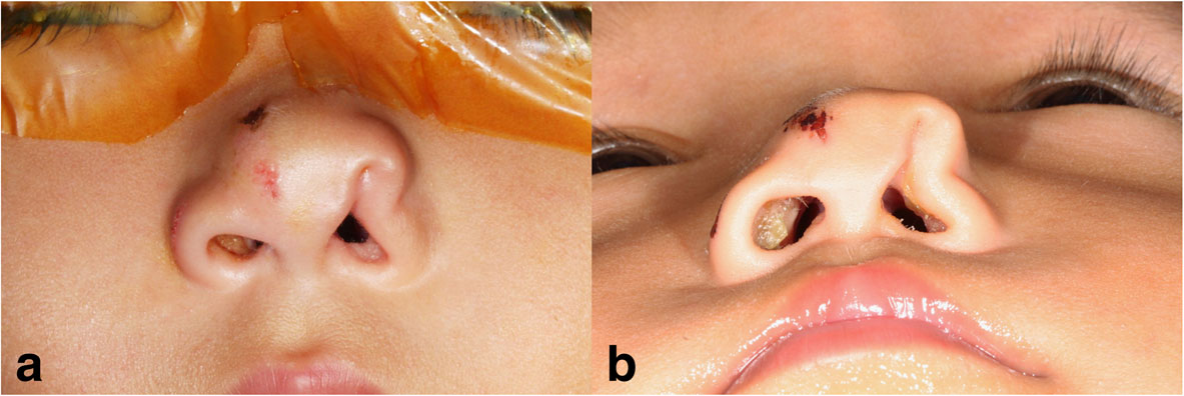
**a** Preoperative frontal view showing accessory nostril above left nostril. **b** Preoperative worm’s eye view

Physical examination showed a left accessory nostril that connected to the normal left nostril. The internal diameter of the accessory opening was measured about 0.2 cm (Fig. 1). Normal ipsilateral nostril was a little smaller than the other side, but the size of the alar base was similar to the right nostril. There was no columellar deviation. The nasal cavity structure was normal. Also, there was no any other deformity on her body. The patient was diagnosed to have a supernumerary nostril.

**Fig. 2:**
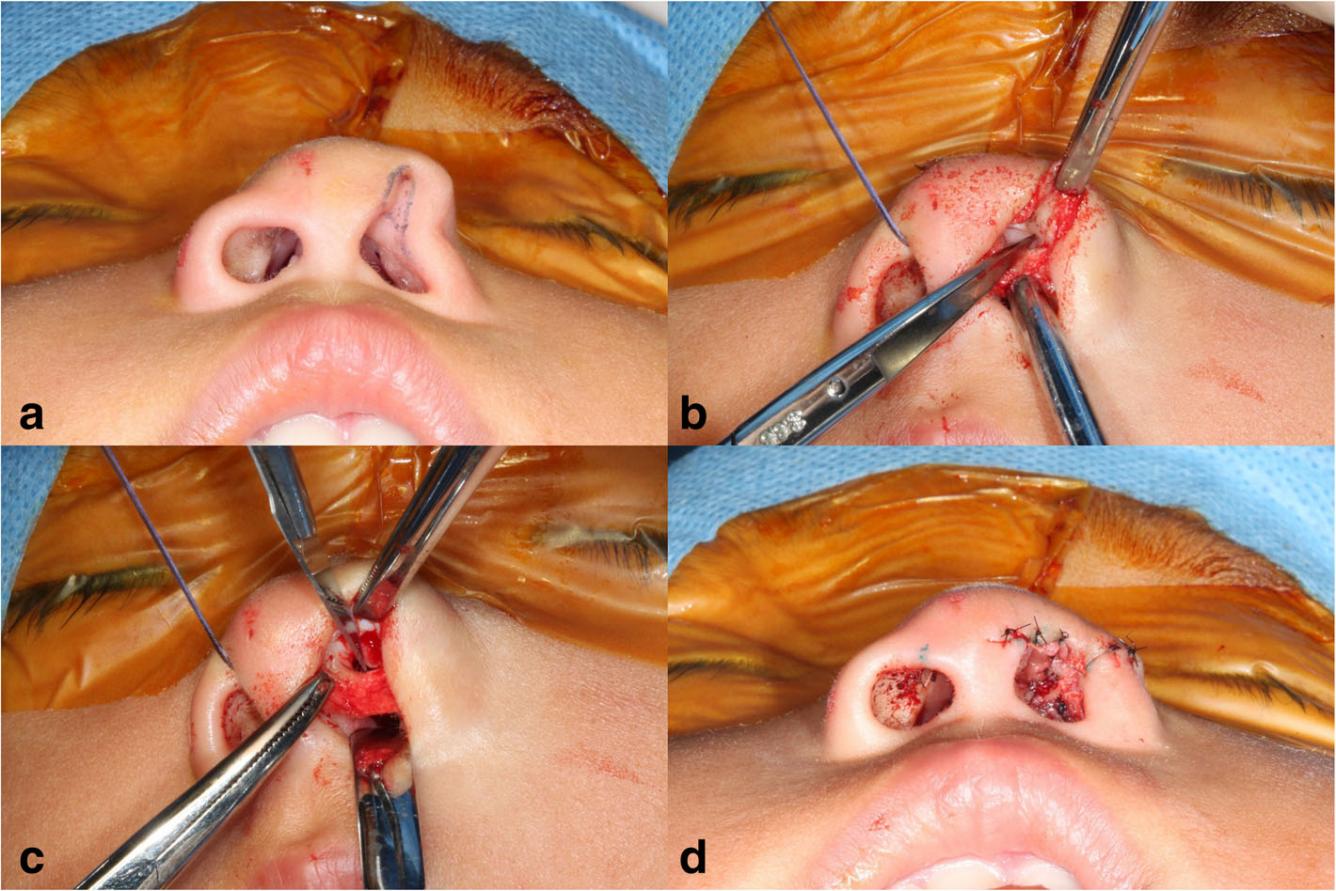
**a** The abnormal structure of the left nostril was drawn using gentian violet solution. **b** Incision and dissection of the fistular tract. **c** The soft tissue across the normal and false nostril was resected. De-epithelialized part of the false and true nostril was attached to each other. **d** Layered suture was applied to the alar rim

Surgery was operated under general anesthesia. First, we drew the abnormal structure around the left nostril by gentian violet solution (Fig. 2a). The incision was performed around the accessory nostril, and then, dissection of the tract was done by using Metzenbaum scissors (Fig. 2b). After opening the nostril, the soft tissue across the normal and false nostril was removed. De-epithelialized part of false and normal nostril was attached to each other (Fig. 2c). A wedge-shaped tissue of the alar side wall was removed. Layered suture of the alar rim was applied (Fig. 2d). Minimal surgical procedure was applied. To consider her growth, rhinoplasty will be left as an option for treatment.

**Fig. 3:**
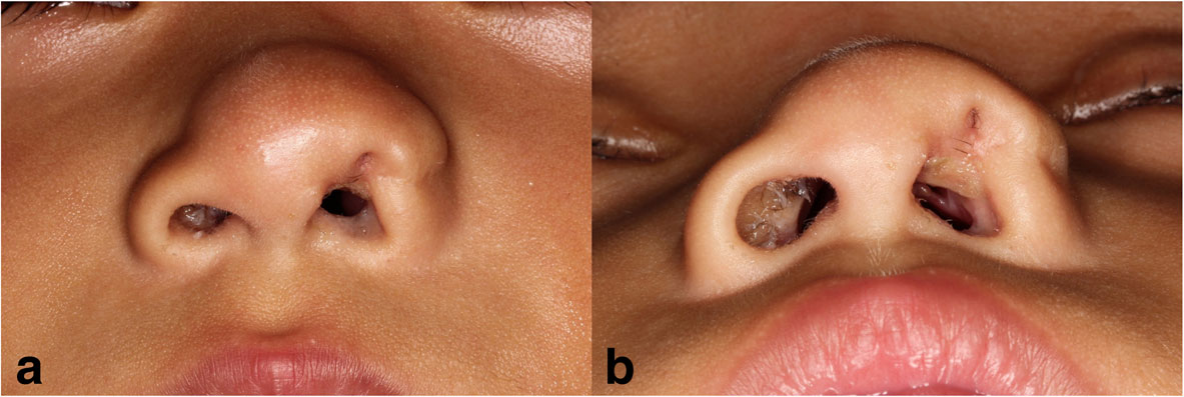
After 1-year follow up. **a** Postoperative frontal view. **b** Postoperative worm’s eye view; irregular-shaped soft tissue remained

**Fig. 4:**
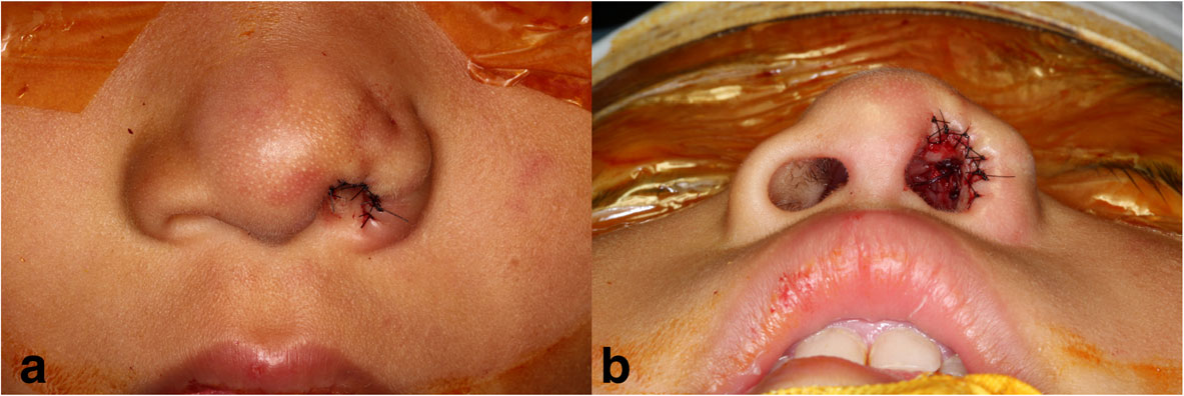
Rhinoplasty was performed. **a** Frontal view. **b** Worm’s eye view

**Fig. 5:**
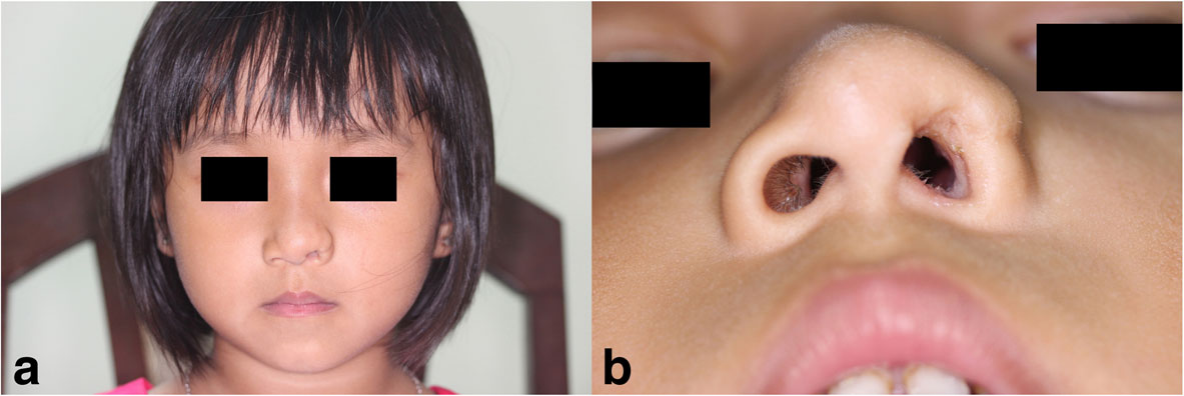
Patient 1 year postoperatively. **a** Frontal view. **b** Worm’s eye view. The nose showed satisfactory cosmetic appearance

After 1-year follow up, the accessory nostril on the left side was totally obstructed but the irregular-shaped soft tissue was remained (Fig. 3). The operated left nostril was larger than the contralateral side. To correct the nostril asymmetry, rhinoplasty was performed (Fig. 4). The patient got cured without special problem. At 1-year postoperative examination, the result and cosmetic appearance was satisfied (Fig. 5).

### Discussion

The nose develops from a frontonasal process in the early fourth week of gestation. The frontonasal process is formed from the forebrain by enlarging and pushing the ectoderm anteriorly and laterally. Nasal placode appears on both sides of the frontonasal process. The nasal placode is the surface ectoderm becoming thicker in two to three cell layers on frontonasal process. In the beginning of the fifth week, the center of the nasal placode sinks to form a nasal groove. As a result, a horseshoe-shaped lateral and medial nasal process appear. As the lateral nasal process and the medial nasal process develop, the nasal groove gets deeper and forms a nasal pit. In the end of the fifth week, the nasal pit gets deeper and becomes nasal sacs. The nasal sacs move into the middle, as maxillary process develops medially. And then, the nasal sacs forms nasal cavity and nostrils [2].

Although the exact pathogenesis of supernumerary nostril has not been revealed, it might cause problem of division of lateral nasal process.

In 1906, Lindsay was first to report the bilateral supernumerary nostril [3]. An accessory nostril was above the normal nostril and connected to the ipsilateral nasal cavity. In 1920, Tawse reported the case of unilateral supernumerary nostril above the right normal nostril [4]. An accessory nostril was connected to the right nasal cavity. In 1962, Erich reported a patient who has two noses [5]. He described that if the position of the accessory olfactory pit is too lateral, the fusion of the laminae is not interfered, which causes the supernumerary nostril. In 1987, Nakamura and Onizuka reported a patient who has divided right alar to medially and laterally [6]. An accessory nostril was located medially. Nakamura and Onizuka hypothesized that supernumerary nostrils resulted from fissuring of the lateral nasal process accidentally. They proposed that the lateral nasal process is divided into two segments and each segment develops into each nostril on the one side of the nose. Also in 1987, Reddy and Rao reported a patient with triple nostrils [7]. An accessory nostril is located below the left normal nostril and connecting into the nasal cavity posteriorly. They assumed that if an accessory olfactory pit appears either above or below the normal location of the placode, consequently, a supernumerary nostril will be formed. In 2009, Kashyap and Khan reported a case of supernumerary nostril on the left side with an accessory alar cartilage [8]. An accessory cavity was smaller than the normal cavity and was not connected with the ipsilateral nasal cavity. Kashyap and Khan proposed that the presence of alar cartilage from the accessory nostril describes the embryological fissuring of the lateral nasal process. In most of the cases, the supernumerary nostril was located on the left side and above the normal nostril. It was either isolated or associated with other congenital anomalies such as cleft lip and palate, naso-ocular cleft, esophageal atresia, and patent ductus arteriosus [9]. Franco et al. reported that 45 % of the patients were related to other congenital anomaly [10].

In the treatment of supernumerary nostril, it is important to remove the accessory nasal tract entirely and preserve the normal nostril. In most cases of supernumerary nostril, fistulectomy and reconstruction with local flaps was performed as a surgical technique. Surgery should be performed as early as possible, because in early age, it is easier to access the deep portion of the nasal cavity, avoid serious affect to nasal cartilage, and avoid deformity around the ala nasi due to fistula. In this case, the accessory opening was obstructed by using simple incision and primary closure without forming local flap. Although the patient had two surgeries to remove the accessory nostril, small and recognizable residual defect on nostril rim of affected side is observed. For the patient’s psychosocial growth, rhinoplasty will be performed after re-evaluating face.

## Conclusions

Supernumerary nostril is a rare congenital deformation of the nose. To our knowledge, this is the second case in our country. In this case, the accessory opening was obstructed by using simple incision and primary closure without forming local flap. And then, rhinoplasty was done. At 12 months follow-up, the patient showed satisfactory result esthetically. In the treatment of supernumerary nostril, it is important to remove the accessory nasal tract entirely and preserve the normal nostril. Early period surgery is important for the patient’s desirable appearance, psychosocial growth, and functional reconstruction.
